# Role of Corn Peptide Powder in Lipopolysaccharide-Induced Inflammatory Responses in 3T3-L1 Adipocytes

**DOI:** 10.3390/nu16121924

**Published:** 2024-06-18

**Authors:** Vijayakumar Mayakrishnan, Dae-Hee Lee, Kee-Hong Kim, Choon Young Kim

**Affiliations:** 1 Research Institute of Human Ecology, Yeungnam University, Gyeongsan 38541, Republic of Korea; drvijay@yu.ac.kr; 2 Sempio Foods Company, 183 Osongsaengmyeong 4ro, Osongeup, Cheongwongun 28156, Republic of Korea; ldaehee@sempio.com; 3 Department of Food Science, Purdue University, West Lafayette, IN 47907, USA; 4 Department of Food and Nutrition, Yeungnam University, Gyeongsan 38541, Republic of Korea

**Keywords:** oxidative stress, inflammation, lipopolysaccharide, obesity, adipocyte, peptide

## Abstract

Corn peptide (CP) is a short, naturally occurring, and physiologically active peptide generated from corn-protease-catalyzed hydrolysis. CP plays a role in preventing obesity-related disorders, but its impact on reducing inflammation is unknown. Hence, this study examined the possible protective effects of corn peptide powder (CPP) against the harmful effects of lipopolysaccharide (LPS), with a particular emphasis on reducing oxidative damage and inflammation in adipocytes. Hence, mature 3T3-L1 adipocytes underwent exposure to 10 ng/mL LPS, with or without CPP (10 and 20 μg/mL). LPS stimulation increased reactive oxygen species and superoxide anion generation. However, this effect was reduced in a dose-dependent manner by pretreatment with CPP. CPP treatment elevated the mRNA expressions of the antioxidant enzymes manganese superoxide dismutase (mnSOD) and glutathione peroxidase 1 (Gpx1) while reducing the mRNA expressions of the cytosolic reactive oxygen species indicators p40 and p67 (NADPH oxidase 2). In addition, CPP inhibited the monocyte chemoattractant protein-1, tumor necrosis factor-alpha, Toll-like receptor 4, and nuclear factor kappa B mRNA expressions induced by LPS. These findings demonstrate that CPP may ameliorate adipocyte dysfunction by suppressing oxidative damage and inflammatory responses through a new mechanism known as Toll-like receptor 4/nuclear factor kappa B-mediated signaling.

## 1. Introduction

Inflammation increases the risk of obesity, type-2 diabetes, cancer, cardiovascular disease, asthma, and inflammatory bowel disease [1,2,3,4]. Globally, a prolonged inflammatory response plays a predominant role in morbidity and mortality, contributing to over 50% of total deaths [5]. The etiology of chronic inflammatory diseases may vary; however, common pathways often lead to pathological abnormalities. Consequently, these common inflammatory pathways offer potential avenues for the development of novel therapeutic interventions. Obesity has been found to be associated with long-lasting, mild inflammation in fat tissue, where there is the increased presence of macrophages and consequent inflammatory reactions [6,7]. Inflammation within the adipose tissue is defined as an increase in macrophage infiltration [8]. Research showed that lipopolysaccharide (LPS) stimulation increased levels of inflammatory adipocytokines, including monocyte chemoattractant protein-1 (MCP-1), interleukin (IL)-6, and tumor necrosis factor-alpha (TNF-α). Nuclear factor kappa B (NF-κB) has been identified as a pivotal player in obesity-related inflammation, with its role centered around the regulation of inflammatory adipocytokines. Recent research suggested that inhibiting adipocyte inflammation through the blockade of NF-κB activation and the production of inflammatory adipocytokines may hold promise for preventing obesity-associated diseases [9].

Despite the availability of many anti-inflammatory medications on the market, these pharmaceutical therapies often cause harmful side effects when used over a long period [10,11]. Nonsteroidal anti-inflammatory drugs, such as aspirin, naproxen, and ibuprofen, are frequently administered for chronic inflammation. They function by blocking the activation of cyclooxygenases involved in the production of proinflammatory substances [12]. However, these medications are associated with significant toxicity and high blood pressure, making them unsuitable for use in older patients with hepatic, renal, and cardiovascular issues [10,13,14,15,16]. Therefore, natural compounds (quercetin, coumarin, luteolin, and baicalein) possessing anti-inflammatory characteristics, which can be incorporated into nutraceutical formulations or functional foods, offer a potentially superior alternative to manufactured medications for the prevention and treatment of inflammatory illnesses. For example, bioactive peptides with diverse immunomodulatory properties have attracted attention as potential therapies for inflammatory conditions. Enzymatic hydrolysis, particularly by digestive enzymes, is the most popular and straightforward technique for generating bioactive peptides because the majority are hidden or encoded in the structure of mature proteins [17]. The oral administration of bioactive peptides produced by the application of gastrointestinal enzymes has been made feasible [18]. Numerous studies have investigated the anti-inflammatory properties of isolated and synthesized peptides. Several research groups have explored the effects of protein hydrolysates containing diverse bioactive peptides [19].

Corn proteins are recognized as significant reservoirs of bioactive peptides. The application of corn protein is limited because of its low solubility in water; however, its solubility can be enhanced through enzymatic hydrolysis. Various methods are available for enhancing the solubility of corn, including acid, alkaline, and enzymatic hydrolysis. Enzymatic hydrolysis is considered to be the most suitable method because of its controllable and gentle reaction conditions, widespread commercial availability, and excellent product quality. However, the conventional enzymatic hydrolysis of proteins has several drawbacks, including inefficient enzyme utilization, low substrate conversion rates, lengthy reaction times, and excessive energy consumption. These limitations can be attributed to the conformation of the substrate protein, which makes it difficult for proteases to hydrolyze peptide bonds. Therefore, the development of more effective enzymatic hydrolysis techniques is highly valuable [20].

Recent studies have explored the use of proteins derived from low-value commercial sources, including rotifer cultures [21], fish byproducts [22], algal waste [23], and the poultry industry [24]. The proteins have been treated with enzymes to produce protein hydrolysates. These hydrolysates show great potential for enhancing the functional properties of proteins. Small peptides have several advantages over proteins, including a lower osmotic pressure, faster absorption, better taste, and reduced antigenicity. In addition, these hydrolysates not only maintain and often improve their biological value but also possess functional properties that surpass those of the original proteins. The hydrolysates also contain bioactive peptides [25].

Small fragments of proteins known as bioactive peptides have been found to have potential health benefits in humans. In recent years, significant discoveries have been made regarding the role of small peptides derived from food proteins [20]. These peptides have been found to have various important functions, such as regulating the autonomic nervous system, activating cellular immunity, improving cardiovascular health, and reducing oxidative stress and the effects of aging. These peptides have also shown a wide range of biological activities, including antioxidative, antihypertensive, immunomodulatory, antimicrobial, and anticancer activities [20]. In addition, studies have shown that dipeptides and tripeptides from the intestinal tract are absorbed more effectively by the body than free amino acids. Functional peptides have a wide range of applications, including their use as additives or texture enhancers in foods and active pharmaceutical ingredients [20,26,27]. Unfortunately, limited information is available on the anti-inflammatory effects of corn protein hydrolysates. This study explored the anti-inflammatory properties of corn protein by examining its anti-inflammatory mechanisms. This investigation of the anti-inflammatory properties of corn protein indicates its value as a powerful natural anti-inflammatory source.

## 2. Results

### 2.1. Determination of Antioxidant Potential of Corn Peptide Powder (CPP)

**Figure 1:**
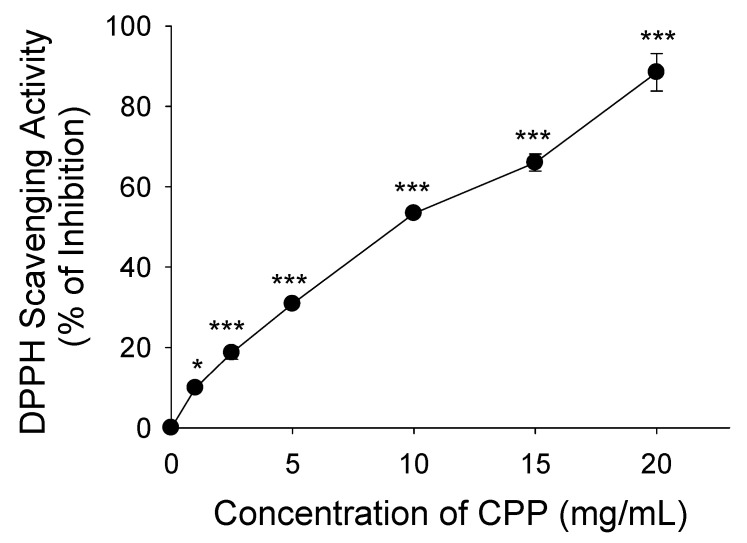
Determination of DPPH radical scavenging activity of CPP. All experiments were conducted in triplicate, and the data are presented as the mean ± standard deviation (*n* = 9) for each tested concentration. * *p* < 0.05; *** *p* < 0.001.

The antioxidant potential of CPP was determined by using its DPPH radical scavenging activity. We measured the ability of CPP to neutralize reactive oxygen species (ROS) and prevent oxidative damage using the 2,2-diphenyl-1-picrylhydrazyl) (DPPH) radical scavenging potential. The antioxidant potential of CPP was calculated as the ascorbic acid equivalent, mgAAE/g (Figure 1). The study results showed the DPPH radical scavenging activity of CPP was dose-dependent, observed at concentrations of 0, 1, 2.5, 5, 10, 15, and 20 mg/mL, with an IC_50_ value of 9.36 mg/mL.

### 2.2. Measurement of Intracellular ROS and Superoxide Anion Production

**Figure 2:**
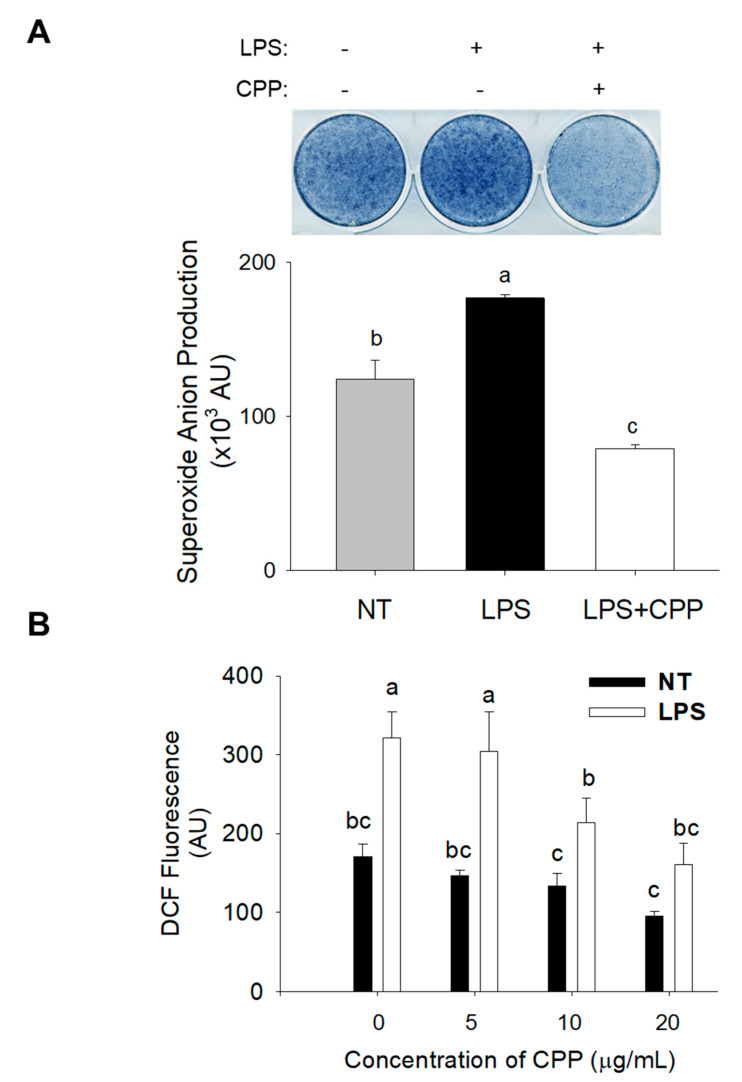
Inhibitory effects of CPP on LPS-induced ROS and superoxide anion levels in mature 3T3-L1 preadipocytes. Changes in (**A**) superoxide anion and (**B**) reactive oxygen species (ROS) levels in mature 3T3-L1 adipocytes. (NT, nontreated control; LPS, lipopolysaccharide; LPS + CPP, lipopolysaccharide + corn peptide powder). Different letters (a, b, and c) above the column indicate significant differences between groups.

ROS play a significant role in promoting the conversion of 3T3-L1 pre-adipocytes into adipocytes. To evaluate ROS levels, particular fluorescent probes, specifically dihydroethidium for superoxide anion and 2′–7′-dichlorofluorescin diacetate for ROS, were used. The findings illustrated in Figure 2A,B demonstrate an increase in the intensity of dihydroethidium fluorescence. To study the potential decrease in superoxide anion and ROS levels induced by CPP, the levels of these components were assessed after treatment with CPP and LPS for 24 h. The findings demonstrated that the administration of CPP at a dose of 20 µg/mL effectively reduced the increase in the superoxide anion levels. Moreover, at concentrations ranging from 0 to 20 µg/mL, CPP considerably (*p* < 0.05) diminished the elevated levels of ROS elicited by LPS. When CPP was administrated at a concentration of 20 µg/mL, it led to a decrease in DCF fluorescence to 180 AU. This was in contrast to the nontreated control group in which LPS treatment induced an increase in DCF fluorescence to 320 AU. These observations indicate that CPP has the potential to mitigate the initial increase in the ROS and superoxide anion levels within 24 h. These results suggest that CPP effectively suppresses LPS-induced ROS production in mature 3T3-L1 adipocytes.

### 2.3. Inhibitory Effect of CPP on ROS production

**Figure 3:**
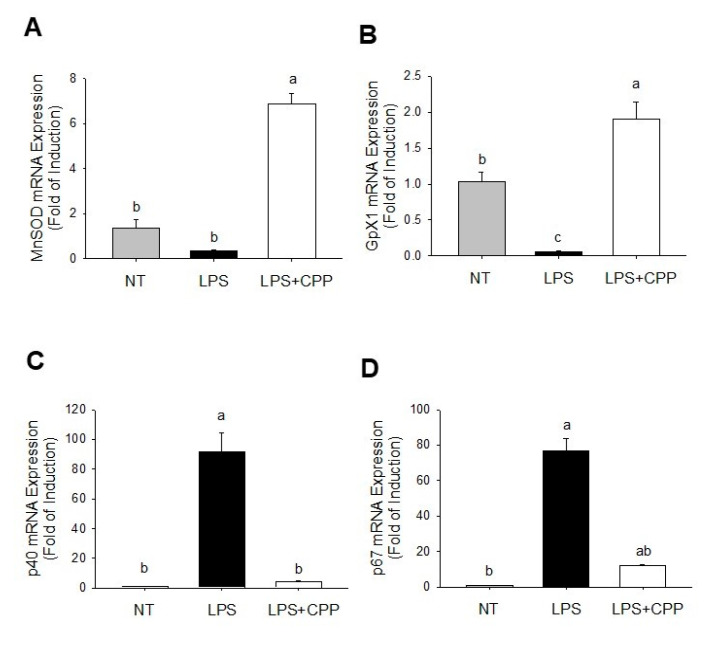
Effects of CPP on mnSOD and Gpx1 mRNA expression and the NOX2 subunits of p40 and p67 mRNA expressions in mature 3T3-L1 adipocytes treated with LPS. Changes in (**A**) MnSOD, (**B**) Gpx1, (**C**) p40, and (**D**) p67 expression levels. (NT, nontreated control; LPS, lipopolysaccharide; LPS+CPP, lipopolysaccharide + corn peptide powder). Different letters (a, b, and c) above the column indicate significant differences between groups.

The effect of CPP treatment on the levels of ROS-related antioxidant enzymes in LPS-stimulated mature adipocytes was assessed using quantitative real-time PCR (qRT-PCR), as illustrated in Figure 3. Our findings revealed a substantial seven-fold increase in the mRNA expression of manganese superoxide dismutase and a two-fold increase in glutathione peroxidase 1 (Gpx1) expression in cells treated with 20 µg/mL CPP, compared with cells treated with LPS (Figure 3A,B). Additionally, CPP treatment at 20 µg/mL led to significant 5-fold and 10-fold decreases in the mRNA expressions of p40 and p67, respectively, when compared with those of LPS-treated cells (Figure 3C,D). These results suggest that the reduction in ROS production is associated with an upregulation in the expression of antioxidant enzymes such as manganese superoxide dismutase and Gpx1, coupled with the downregulation of NADPH oxidase 2 enzymes such as p40 and p67.

### 2.4. Effects of CPP on LPS-Induced TNF-α and MCP1 Expression

**Figure 4:**
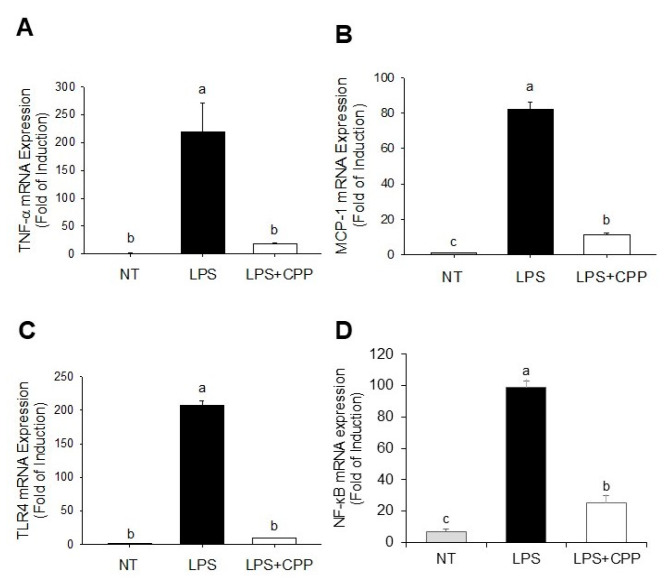
Inhibitory effects of CPP on MCP-1, TNF-α, and TLR4 mRNA expression levels in mature 3T3-L1 adipocytes treated with LPS. Changes in (**A**) MCP-1, (**B**) TNF-α, (**C**) TLR4, and (**D**) NF-κB expression levels. (NT, nontreated control; LPS, lipopolysaccharide; LPS + CPP, lipopolysaccharide + corn peptide powder). Different letters (a, b, and c) above the column indicate significant differences between groups.

MCP1 and TNF-α are essential mediators in the progression of chronic inflammation in adipose tissue. To examine the potential anti-inflammatory properties of CPP, we employed the qRT-PCR method to measure the levels of gene expression of TNF-α and MCP1. Figure 4A,B clearly show that the gene expressions of TNF-α and MCP1 were drastically increased by LPS in comparison to those of the control group. Nevertheless, the CPP treatment successfully suppressed the increases in TNF-α and MCP1 gene expressions generated by LPS. The results of the present study showed a significant increase in the amount of NF-κB gene expression in 3T3-l1 cells after exposure to LPS alone. However, CPP reduced LPS-induced NF-κB expression in 3T3-L1 cells.

### 2.5. CPP Suppresses the Expression of the Toll-like Receptor 4 (TLR4) Gene Triggered by LPS

TLR4 is the principal receptor responsible for LPS recognition. We further evaluated the anti-inflammatory mechanism by which CPP affects TLR4 expression. The upregulation of TLR4 expression was significantly induced by LPS compared with that in the untreated group, as illustrated in Figure 4C. The LPS-induced upregulation of TLR4 expression was effectively inhibited by CPP.

## 3. Discussion

Oxidative stress occurs when there is an imbalance in redox reactions that favors pro-oxidants, leading to the overproduction of ROS. ROS act as primary catalysts, initiating bimolecular oxidation and causing oxidative stress [28,29]. Antioxidants mitigate the effect of free radicals by directly reacting with, competing with, or neutralizing substances that use molecular oxygen (O_2_) as their final electron acceptor. Therefore, molecular oxygen functions as a thermodynamic reservoir [30]. Numerous commercially available synthetic antioxidant agents have been reported to exhibit toxicity and carcinogenicity, highlighting the natural antioxidants from medicinal plants as preferable alternatives for combating oxidative damage [31]. Herbal medicines are valuable therapeutic agents for managing and preventing the degenerative diseases associated with oxidative stress. In this study, CPP demonstrated noteworthy free radical scavenging activity.

The DPPH radical scavenging activity of CPP was directly correlated with the concentration. CPP at concentrations of 0–20 mg/mL exhibited antioxidant activity against DPPH. The antioxidant activity of these CPPs was significantly higher than that previously reported [32,33]. CPP caused a dose-dependent reduction in capacity, with the highest level achieved at a concentration of 20 mg/mL. Similarly, the bioactive peptide A-10-F produced from milk showed scavenging activity against DPPH radicals, which increased at higher concentrations [34]. The presence of amino acids involved in free radical deactivation may contribute to the significant antioxidant activity observed in the crude protein. The quenching of reactive oxygen/nitrogen species can be achieved by antioxidant peptide molecules via two pathways: hydrogen atom transfer and single-electron transfer reactions. In biological systems, Met is a crucial free radical scavenger. This can be attributed to the Met radical, which is highly susceptible to oxidation and has the potential to neutralize free radicals before they harm the other amino acid residues that are essential for protein structure and function. To gain further insight into the structural properties of antioxidant peptides derived from corn protein hydrolysates, it is necessary to purify the active peptides and analyze their amino acid sequences.

ROS are produced by the mitochondria, which are also an important source of oxygen metabolism in fat cells. Increased ROS levels were detected in the early stages of adipocyte differentiation in both 3T3-L1 cell lines, suggesting that ROS production is necessary for adipocyte differentiation. ROS can be produced by mitochondrial complex III and control the differentiation of primary human mesenchymal stem cells into adipocytes. This differentiation process is primarily influenced by mammalian target of rapamycin complex 1 signaling [35]. Hypoxia (2% oxygen) promotes development in adipose-derived stem cells by generating mitochondrial ROS. Consequently, we investigated the potential correlation of MT3 and ROS levels with adipocyte development [36]. Moreover, excessive ROS levels can modify adipocytes and lipids, resulting in adipocyte dysfunction characterized by altered cell signaling, impaired energy metabolism, and inflammation [37].

Antioxidant peptides can eliminate ROS through several mechanisms, such as donating electrons or hydrogen atoms, chelating metal ions, scavenging various types of ROS, modulating the activity of endogenous antioxidant enzymes, inhibiting enzymes producing ROS, and repairing oxidatively damaged molecules. Various methods have been used to assess the antioxidant activity of walnut peptides through the regulation of ROS levels. Ren et al. assessed the impact of Manchurian walnut hydrolyzed peptide (MWHP) on the production of ROS and glutathione peroxidase in PC12 cells induced by H_2_O_2_. The results indicated that in comparison to the positive control group (H_2_O_2_ injury group), ROS production was effectively suppressed in the three MWHP groups, especially in the <3 kDa MWHP group [38]. Jahanbani et al. used ROS to evaluate the effects of WPH on human breast (MDA-MB231) and colon (HT-29) cancer cell lines. Their findings revealed that peptides derived from walnuts demonstrated significant antioxidant effects on these cells [39].

It is widely acknowledged that inflammation and the recurrence of chronic disease and aging are significantly influenced by the cellular antioxidant defense system. Research has indicated that the activities of superoxide dismutase 2 (SOD2), GPX1, and catalase inhibit the production of ROS, which in turn causes oxidative damage to proteins, lipids, and DNA [40]. Recent studies have highlighted the potential of endogenous antioxidant enzymes such as SOD2 and Gpx1 as markers for the progression of ROS and the development of obesity [41]. In light of this, the current study aimed to examine the levels of oxidative-stress-related genes, including SOD2 and Gpx1, as the activity of the main antioxidant enzymes found in cells is typically upregulated in response to extreme oxidative stress [42]. Our results showed that LPS-induced oxidative stress led to the downregulation of SOD2 and Gpx1 in 3T3-L1 adipocytes compared with untreated cells. This downregulation suggests that the cells promptly activate their internal antioxidant systems to counteract stress. CPP treatment resulted in different response patterns in the expression of genes encoding antioxidant enzymes. CPP upregulated SOD2 and Gpx1, indicating its protective effect against LPS-induced oxidative stress. According to certain authors [43], peptides derived from food have protective effects by activating the production of proteins that safeguard cells from damage caused by oxidative stress. It is possible that CPP directly scavenges or inactivates radicals, thereby mitigating the activation of internal antioxidant defense mechanisms. These findings suggest that the compounds in CPP may prevent oxidative damage by scavenging intracellular ROS and enhancing the expression of the antioxidant genes *SOD2* and *Gpx1*.

The NADPH oxidase components in adipose tissue are associated with obesity-related ROS production. The expression levels of NADPH oxidase components in adipose tissue are elevated in obese mice [44]. Previously, it was reported that the mRNA levels of NADPH oxidase subunits, including p40^phox^, p47^phox^, p22^phox^, and gp91^phox^, were significantly increased in the adipose tissue of genetically obese mice [45]. NADPH oxidase partially mediates the increased ROS production in the adipose tissue of mice with obesity and the insulin resistance induced by a high-fat diet [46]. In this study, we found that adipocyte-specific CPP downregulates two cytosolic NADPH oxidase components, p40^phox^ and p67^phox^. p40^phox^ is necessary for the activation of NADPH oxidase through the transportation of the cytosolic complex to the cell membrane. Given its structural similarity to p67^phox^, p40^phox^ could serve as a substitute for p67^phox^ to sustain ROS generation [47]. Furthermore, the absence of p47phox in mice protects from the infiltration of macrophages into adipose tissue, the production of TNFα and IL-6 in adipose tissue, and the development of systemic insulin resistance caused by a high-fat diet [48]. The inhibition of p40^phox^ and p67^phox^ consequently impairs NADPH oxidase activation, leading to a reduction in ROS production [49]. Our findings demonstrate that CPP significantly inhibits NADPH oxidase activity, resulting in reduced ROS levels in adipocytes treated with CPP. This reduction in ROS levels may also be attributed to the upregulation of oxidative defense mechanisms. 

Numerous studies have demonstrated the anti-inflammatory properties of peptides [50]. Our results showed that LPS-induced inflammation was successfully inhibited by CPP in mature 3T3-L1 adipocytes. The generation of inflammatory responses in the fat tissue is a hallmark of obesity. The accumulated evidence demonstrates an increase in MCP-1 and TNF-α levels in the adipose tissues of individuals with obesity [51]. MCP-1, which is mostly produced and released by adipocytes, has been documented to play an essential role in attracting macrophages to fat tissues and promoting impaired insulin sensitivity [52]. Adipokines and cytokines contribute to chronic inflammation, which in turn causes the emergence of obesity-related metabolic disorders. Previous studies have demonstrated the clinical importance of preventing the release of inflammatory cytokines in the treatment of metabolic problems associated with obesity [41]. The current investigation revealed that LPS markedly increased the expressions of TNF-α and MCP-1. These findings imply that CPP has anti-inflammatory properties in LPS-activated 3T3-L1 adipocytes. Consistent with previous research, the secretion of proinflammatory cytokines was substantially reduced in the peptide-treated group compared with that in the LPS model group [53]. The inhibitory effects of peptide on inflammatory factor activity in RAW264.7 cells might be the result of in situ enzymatic hydrolysis. In general, peptides mitigate inflammatory responses by modulating the concentration of inflammatory factors [54].

Numerous genes that control inflammation, cell survival, proliferation, and migration are induced or suppressed by the activation of these transcription factors. When NF-κB transcriptional activity is blocked, proinflammatory mediators and cytokines, including TNF-α, IL-1β, and IL-6, are not expressed. This highly regulated process can lead to multiple organ failure, autoimmune disorders, acute or chronic inflammatory diseases, encouraging tumor cell proliferation and even malignancies if it is not under control [55,56]. NF-κB, a transcription factor, plays a crucial function in regulating MCP-1 and TNF-α production [57]. Previous research indicated that the stimulation of adipocytes by LPS triggers the movements of NF-κB and p65 toward the nucleus, regulating MCP-1 and TNF-α. Our results indicate that CPP significantly inhibits the expression of MCP-1 and TNF-α induced by LPS. TLR4 serves as the primary receptor for LPS recognition, with studies revealing that LPS triggers TLR4 signaling pathway activation, subsequently inducing TNF-α [58]. Furthermore, we examined the mechanism of action of CPP in reducing inflammation and examined whether this involves the inhibition of TLR4 signaling. The findings showed that CPP decreases the TLR4 expression upregulated by LPS, indicating that CPP suppresses the TNF-α activation generated by LPS by inhibiting TLR4 signaling. Moreover, the inhibitory effects of a peptide found in the albumen of eggs derived from ovotransferrin were elucidated previously, and the peptide demonstrated anti-inflammatory activity by inhibiting NF-κB-related p50 and p65 [59].

CPP, which is derived from corn protein hydrolysate, shows promise as a bioactive compound with anti-inflammatory properties. The 3T3-L1 adipocyte cell model serves as a representative system for investigating the effects of CPP on adipose tissue, which is a key player in metabolic health. The induction of inflammatory responses by LPS mimics the conditions associated with obesity-related inflammation. The findings of this study provide insight into the molecular mechanisms underlying the anti-inflammatory effects of CPP on adipocytes, shedding light on its potential role in mitigating adipose tissue inflammation. Understanding how CPP modulates inflammatory pathways in adipocytes may contribute to the development of nutritional strategies or functional foods aimed at alleviating the inflammation associated with obesity and metabolic syndrome, thus offering potential therapeutic benefits for individuals at risk of related health complications.

## 4. Materials and Methods

### 4.1. Reagents

Butylated hydroxytoluene, 2′,7′–dichlorofluorescin diacetate, DMSO, 3-(4,5-dimethylthiazol-2-yl)-2,5-diphenyltetrazolium bromide, and nitroblue tetrazolium (NBT) were purchased from Sigma-Aldrich (St. Louis, MO, USA). LPS and DPPH were purchased from Cayman Chemicals (Ann Arbor, MI, USA). Dulbecco’s modified Eagle’s medium (DMEM), 0.25% trypsin-EDTA, sodium pyruvate, and penicillin/streptomycin were obtained from Thermo Fisher Scientific (Waltham, MA, USA). Fetal calf serum and fetal bovine serum (FBS) were obtained from PAA Laboratories (Pasching, Austria). PCR primers were purchased from Invitrogen (Carlsbad, CA, USA). All the other chemicals were of analytical grade.

### 4.2. Preparation of CPP

CPP was acquired from Sempio Foods Company (Incheon, Korea). Corn proteins were hydrolyzed using alcalase (U/g), protamex (U/g), and flavourzyme (U/g) from Novo Nordisk’s Enzyme Business (Fuglebakken, Denmark), as provided by the Sempio Foods Company. The nutrient and amino acid compositions are listed in Table 1.

### 4.3. DPPH Radical Scavenging Assay

The DPPH free radical assay has been extensively used to assess antioxidant activity. The DPPH radical, when reduced and converted to DPPH-H, initially exhibits a dark purple coloration in solution [60]. A lower IC_50_ value signifies greater efficacy in scavenging DPPH free radicals [61]. DPPH radical scavenging activity was evaluated using the procedure described by Choi et al. [62]. In summary, CPP was diluted to quantities ranging from 0 to 20 mg/mL in distilled water. A 150 μM DPPH solution was added to each sample, the reaction was allowed to occur at room temperature for a duration of 30 min, and the absorbance was measured at 517 nm using an automated microplate reader (EnSpire^TM^ multimode plate reader, PerkinElmer, Waltham, MA, USA). To produce a calibration curve, various concentrations of standard ascorbic acid were prepared from the stock solutions (0, 0.125, 0.25, 0.5, and 1.0 mg/mL). A volume of 1 mL was transferred from each standard ascorbic acid solution into five distinct 25 mL volumetric flask, followed by 4 mL of methanol and 2 mL of DPPH solution, and the solution was incubated at room temperature for a duration 30 min. The calibration curve was constructed by measuring the absorbance at 517 nm. The equation provided below was used to calculate the % inhibition of the CPP in the presence of DPPH.

% inhibition *=* Ac − Ao/Ac × 100

Ac represents the absorbance of the pure, oxidized form of DPPH (used as a control), whereas Ao refers to the absorbance of the CPP measured 30 min after reacting with DPPH. The experiment was conducted in triplicate.

### 4.4. Cell Culture and LPS Treatment

The 3T3-L1 cells were obtained from the American Type Culture Collection (ATCC CL-173) and cultivated in an incubator at a temperature of 37 °C, with an atmosphere consisting of 5% CO_2_, and 95% humidified air. The cells were cultured in DMEM containing 25 mM HEPES, 1% penicillin–streptomycin (100 U/mL; 100 μg/mL), and 10% calf serum. Cell differentiation began two days after reaching full capacity (day 0) in DMEM supplemented with 10% FBS, 0.25 μM dexamethasone, 0.5 mM 3-isobutyl-1-methylxanthine, and 5 μg/mL insulin for a duration of 48 h. Afterward, the cells were cultured in a solution containing 10% FBS and DMEM supplemented with insulin for 72 h. The culture medium containing 10% FBS/DMEM was refreshed every two days. The cells underwent a 24 h pretreatment with a medium containing CPP (10 and 20 μg/mL) in the presence or absence of LPS (10 ng/mL) on day 8 of differentiation. The cells and medium were collected in tubes and preserved at a temperature of −80 °C until further experiments.

### 4.5. Quantification of Intracellular ROS Generation

In order to evaluate the effect of CPP on the generation of ROS inside cells after treatment, the liquid in the culture dish was removed, PBS was added, and the cells were exposed to the 2′-7′-dichlorofluorescin diacetate dye at a concentration of 10 μM for 30 min. Fluorescence emission was detected at 530 nm with an excitation wavelength of 485 nm. The fluorescence readings were used to quantify the degree of ROS, which represents the percentage of cellular oxidative stress compared with untreated control cells (cells treated with the vehicle instead of the preparation in the same volume).

### 4.6. Determination of Superoxide Anion (O_2_^• −^) Production

The NBT reduction test was used to evaluate the inhibitory effect of CPP on the generation of O2^• −^ in mature 3T3-L1 cells treated with LPS. Briefly, fully developed 3T3-L1 adipocytes were exposed to 20 µg/mL CPP together with 10 ng/mL LPS in a 10% FBS-DMEM medium for 24 h. The NBT solution, with a concentration of 0.5 mg/mL, was introduced into each well and kept at a temperature of 37 °C in an environment with 5% CO_2_ for a duration of 4 h. The blue formazan crystals produced were observed and disintegrated using a lysis buffer containing DMSO and 2 M KOH in a 1:1 ratio. Formazan formation was quantified at 570 nm using a microplate reader (Beckman Coulter, Brea, CA, USA).

### 4.7. qRT-PCR

miRNA extraction was performed using a TriZol isolation kit (Invitrogen). In summary, total RNA was subjected to reverse transcription using a Reverse Transcription Kit (Applied Biosystems Inc., Foster City, CA, USA) following the manufacturer’s instructions. The synthesized cDNA was analyzed using quantitative RT-PCR using a StepOne real-time PCR instrument (Applied Biosystems Inc.). The primers used are listed in Table 2. Gene expression was normalized using the delta Ct method and referencing endogenous β-actin expression. The results are presented as relative fold induction. β-actin was used as a positive control.

### 4.8. Statistical Analysis

All measurements were conducted in three independent experiments, each with at least triplicate samples. Data are expressed as mean ± standard deviation (SD). Statistical analyses were performed using SAS version 9.4, Cary, NC, USA). Dunnett’s test was used for statistical comparison of DPPH activity. For multiple variable comparisons, the experimental data were subjected to a one-way analysis of variance (ANOVA) followed by Bonferroni’s multiple comparison test. Significance was attributed to differences between the mean values at *p* < 0.05.

## 5. Conclusions

In conclusion, these results indicate that CPP possesses antioxidant and anti-inflammatory properties against the LPS-induced dysfunction of mature 3T3-L1 adipocytes, as demonstrated by scavenging intracellular ROS and enhancing the expression of antioxidant defense enzymes, including SOD2, Gpx1, p40, and p67. Additionally, CPP treatment led to a decrease in MCP-1 and TNF-α levels through the TLR4/NF-κB-mediated signaling. These results demonstrated that CPP is linked to ROS inhibition and proinflammatory cytokine suppression in inflamed mature adipocytes. Further experiments at the biochemical and molecular levels are recommended for confirmation in future research.

## Figures and Tables

**Table 1:** The nutrient compositions of corn peptide powder (CPP).

				(g/100 g CPP)	
Protein				54.00	
	**Essential** (g/100 g protein)		**Nonessential** (g/100 g protein)		
	L-Leucine	11.51	L-Glutamic acid	15.21	
	L-Phenylalanine	3.02	L-Alanine	7.41	
	L-Valine	2.98	L-Proline	5.27	
	L-Isoleucine	2.80	L-Aspartic acid	4.72	
	L-Methionine	1.57	L-Serine	2.66	
	L-Threonine	2.44	L-Glycine	1.61	
	L-Histidine	1.19	L-Arginine	0.34	
	L-Lysine	1.19	L-Cysteine	0.00	
	L-Tryptophan	0.49	L-Tyrosine	0.00	
Carbohydrate				21.00	
Fat				0.00	
Fiber				5.00	
Ash				8.00	
Moisture				12.00	

**Table 2:** Primer sequences for RT-PCR amplification.

Gene	Forward (5′-3′)	Reverse (5′-3′)
MnSOD	GGCCAAGGGAGATGTTACAACT	CCCCCACCATTGAACTTCAG
Gpx1	CTCGGTTTCCCGTGCAAT	GACGTACTTGAGGGAATTCAGAATC
P40	GCACGCCCCTGTTCAAAG	TGGTAGCTAAGGGCAATGTCTTC
P67	CTGCCTGACTCTGTGGTGTGA	CCCTTTGTATGGGTTCATCAAT
TN-α	GGG ACA GTG ACC TGG ACT GT	AGG CTG TGC ATT GCA CCT CA
MCP1	CCACTCACCTGCTGCTACTCAT	AGCTCTCCAGCCTACTCATTGG
TLR4	ACCTGGCTGGTTTACACGTC	GTGCCAGAGACATTGCAGAA
NF-κB	GGAGCACAGATACCACCAAGAC	CTCAGCCTCATAGAAGCCATCC
β-actin	TCCTATGTGGGTGACGAGGC	CATGGCTGGGGTGTTGAAGG

## Data Availability

The original contributions presented in the study are included in the article, further inquiries can be directed to the corresponding author/s.
